# Supplementary material for: AnnSQL: a Python SQL-based package for fast large-scale single-cell genomics analysis using minimal computational resources
Source: Bioinform Adv. 2025 May 5;5(1):vbaf105. doi: 10.1093/bioadv/vbaf105 (PMC12098940; doi:10.1093/bioadv/vbaf105)
Supplement: vbaf105_Supplementary_Data [file vbaf105_supplementary_data.zip › AnnSQL Supplemental Text.docx]

**
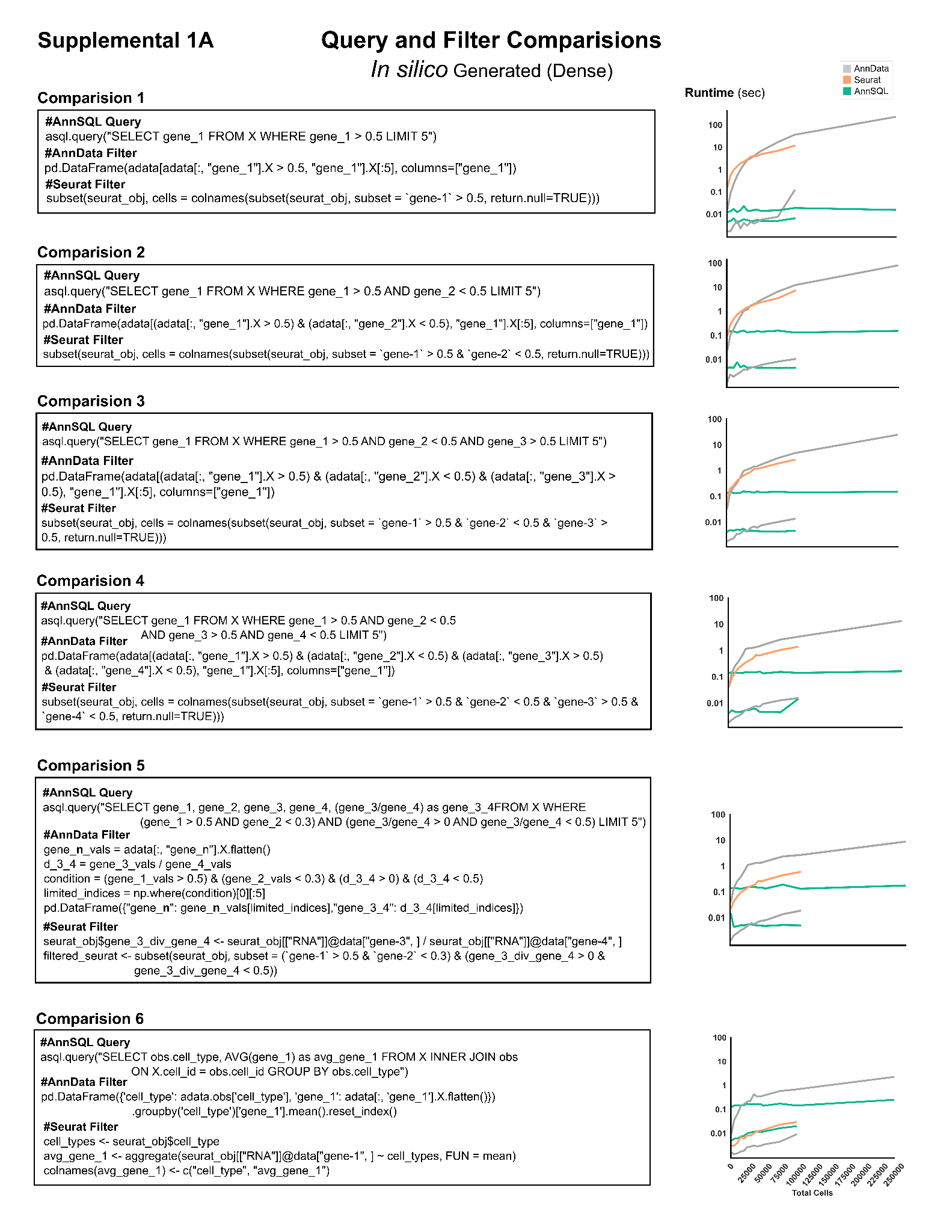
**

Runtime comparisons for six equivalent operations (rows) performed using AnnData versus AnnSQL (summarized

in Figure 1D). Left, operation-specific calls. Right, runtime comparisons for on-disk and in-memory modes.

**Supplemental 2**


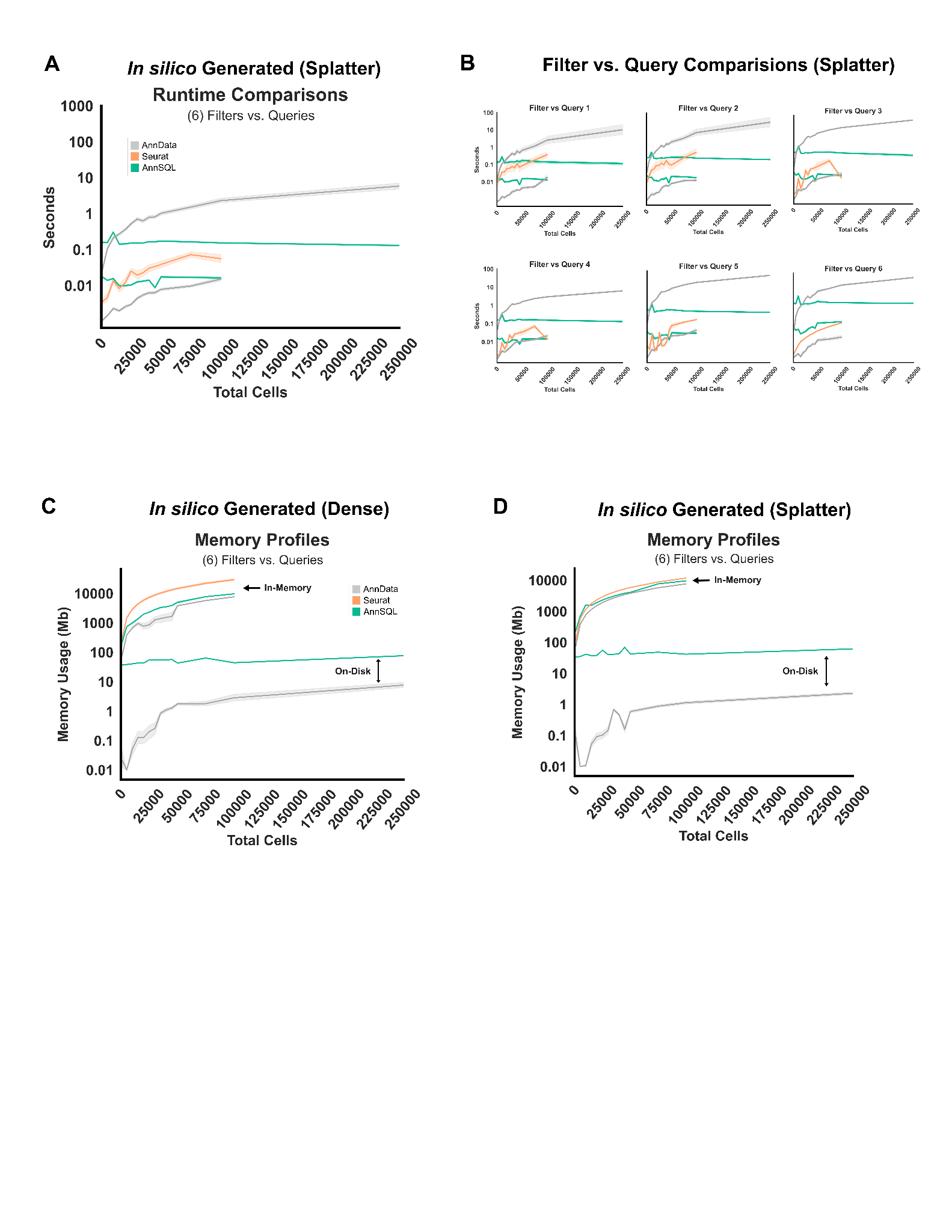


A.) *In silico* runtime analysis of libraries generated using the Splatter sc/nRNA-seq simulator tool. Filter and query comparisons are identical to **Supplemental 1A**. B.) Individual runtime profiles across library sizes for individual filters 1-6 of Splatter generated data. C.) Memory consumption of dense in-silico generated while running query and filter comparisons. D.) Same as C but with Splatter (sparse) generated data. For each figure, filters and queries are repeated six times for each of the six queries and filters. Shading represents 95% confidence intervals.
