# Supplementary material for: AnnSQL: a Python SQL-based package for fast large-scale single-cell genomics analysis using minimal computational resources
Source: Bioinform Adv. 2025 May 5;5(1):vbaf105. doi: 10.1093/bioadv/vbaf105 (PMC12098940; doi:10.1093/bioadv/vbaf105)
Supplement: vbaf105_Supplementary_Data [file vbaf105_supplementary_data.zip › Supplemental 1.pdf]

Supplemental 1A

Query and Filter Comparisions

In silico Generated (Dense)

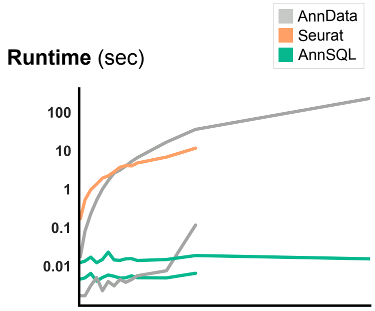

Comparision 1

#AnnSQL Query

```
asql.query("SELECT gene_1 FROM X WHERE gene_1 > 0.5 LIMIT 5")
```

#AnnData Filter

```
pd.DataFrame(adata[adata[:, "gene_1"].X > 0.5, "gene_1"].X[:5], columns=["gene_1"])
```

#Seurat Filter

```
subset(seurat_obj, cells = colnames(subset(seurat_obj, subset = `gene-1` > 0.5, return.null=TRUE)))
```

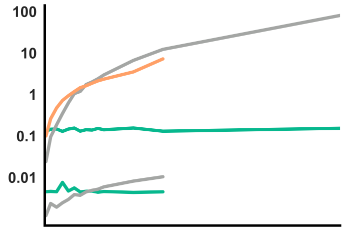

Comparision 2

#AnnSQL Query

```
asql.query("SELECT gene_1 FROM X WHERE gene_1 > 0.5 AND gene_2 < 0.5 LIMIT 5")
```

#AnnData Filter

```
pd.DataFrame(adata[(adata[:, "gene_1"].X > 0.5) & (adata[:, "gene_2"].X < 0.5), "gene_1"].X[:5], columns=["gene_1"])
```

#Seurat Filter

```
subset(seurat_obj, cells = colnames(subset(seurat_obj, subset = `gene-1` > 0.5 & `gene-2` < 0.5, return.null=TRUE)))
```

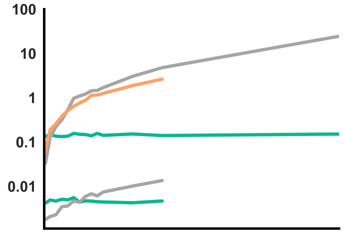

Comparision 3

#AnnSQL Query

```
asql.query("SELECT gene_1 FROM X WHERE gene_1 > 0.5 AND gene_2 < 0.5 AND gene_3 > 0.5 LIMIT 5")
```

#AnnData Filter

```
pd.DataFrame(adata[(adata[:, "gene_1"].X > 0.5) & (adata[:, "gene_2"].X < 0.5) & (adata[:, "gene_3"].X >
0.5), "gene_1"].X[:5], columns=["gene_1"])
```

#Seurat Filter

```
subset(seurat_obj, cells = colnames(subset(seurat_obj, subset = `gene-1` > 0.5 & `gene-2` < 0.5 & `gene-3` >
0.5, return.null=TRUE)))
```

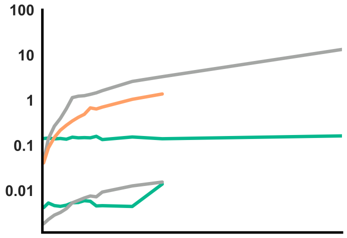

Comparision 4

#AnnSQL Query

```
asql.query("SELECT gene_1 FROM X WHERE gene_1 > 0.5 AND gene_2 < 0.5
            AND gene_3 > 0.5 AND gene_4 < 0.5 LIMIT 5")
```

#AnnData Filter

```
pd.DataFrame(adata[(adata[:, "gene_1"].X > 0.5) & (adata[:, "gene_2"].X < 0.5) & (adata[:, "gene_3"].X > 0.5)
 & (adata[:, "gene_4"].X < 0.5), "gene_1"].X[:5], columns=["gene_1"])
```

#Seurat Filter

```
subset(seurat_obj, cells = colnames(subset(seurat_obj, subset = `gene-1` > 0.5 & `gene-2` < 0.5 & `gene-3` > 0.5 &
`gene-4` < 0.5, return.null=TRUE)))
```

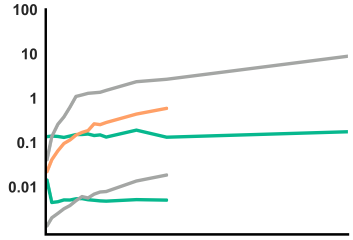

Comparision 5

#AnnSQL Query

```
asql.query("SELECT gene_1, gene_2, gene_3, gene_4, (gene_3/gene_4) as gene_3_4FROM X WHERE
            (gene_1 > 0.5 AND gene_2 < 0.3) AND (gene_3/gene_4 > 0 AND gene_3/gene_4 < 0.5) LIMIT 5")
```

#AnnData Filter

```
gene_n_vals = adata[:, "gene_n"].X.flatten()
d_3_4 = gene_3_vals / gene_4_vals
condition = (gene_1_vals > 0.5) & (gene_2_vals < 0.3) & (d_3_4 > 0) & (d_3_4 < 0.5)
limited_indices = np.where(condition)[0][:5]
pd.DataFrame({"gene_n": gene_n_vals[limited_indices],"gene_3_4": d_3_4[limited_indices]})
```

#Seurat Filter

```
seurat_obj$gene_3_div_gene_4 <- seurat_obj[["RNA"]]@data["gene-3", ] / seurat_obj[["RNA"]]@data["gene-4", ]
filtered_seurat <- subset(seurat_obj, subset = (`gene-1` > 0.5 & `gene-2` < 0.3) & (gene_3_div_gene_4 > 0 &
                  gene_3_div_gene_4 < 0.5))
```

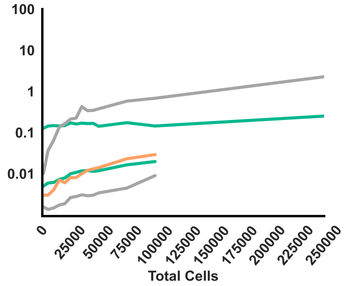

Comparision 6

#AnnSQL Query

```
asql.query("SELECT obs.cell_type, AVG(gene_1) as avg_gene_1 FROM X INNER JOIN obs
            ON X.cell_id = obs.cell_id GROUP BY obs.cell_type")
```

#AnnData Filter

```
pd.DataFrame({'cell_type': adata.obs['cell_type'], 'gene_1': adata[:, 'gene_1'].X.flatten()})
            .groupby('cell_type')['gene_1'].mean().reset_index()
```

#Seurat Filter

```
cell_types <- seurat_obj$cell_type
avg_gene_1 <- aggregate(seurat_obj[["RNA"]]@data["gene-1", ] ~ cell_types, FUN = mean)
colnames(avg_gene_1) <- c("cell_type", "avg_gene_1")
```
