# Supplementary figures and images for: AnnSQL: a Python SQL-based package for fast large-scale single-cell genomics analysis using minimal computational resources
Source: Bioinform Adv. 2025 May 5;5(1):vbaf105. doi: 10.1093/bioadv/vbaf105 (PMC12098940; doi:10.1093/bioadv/vbaf105)

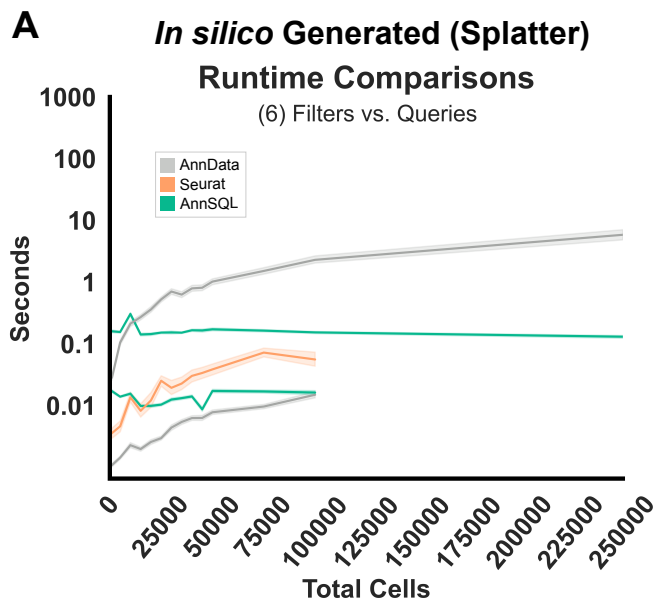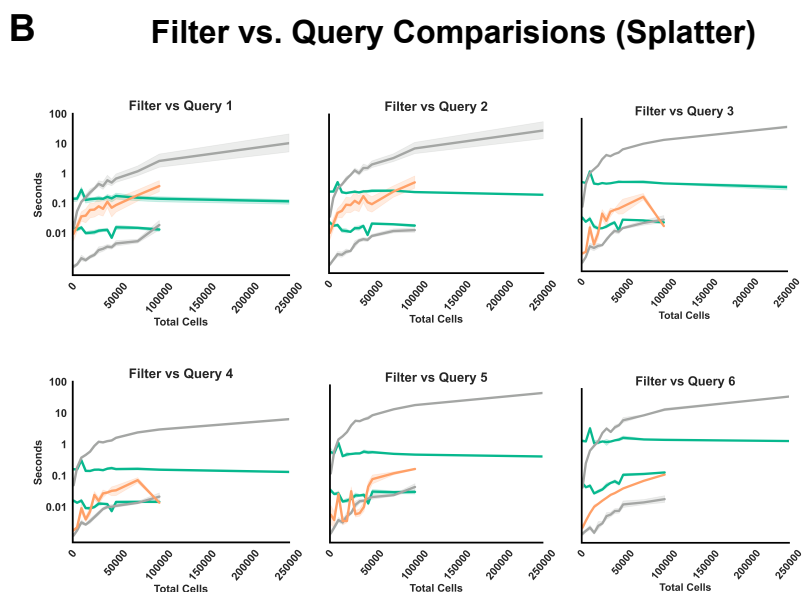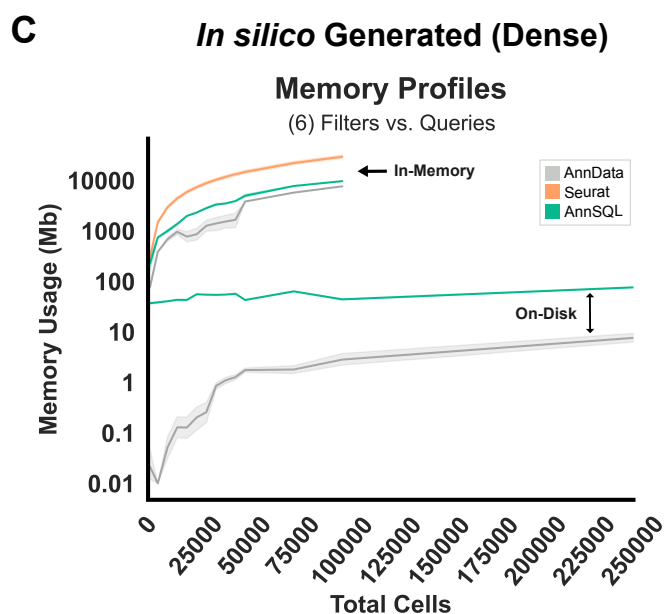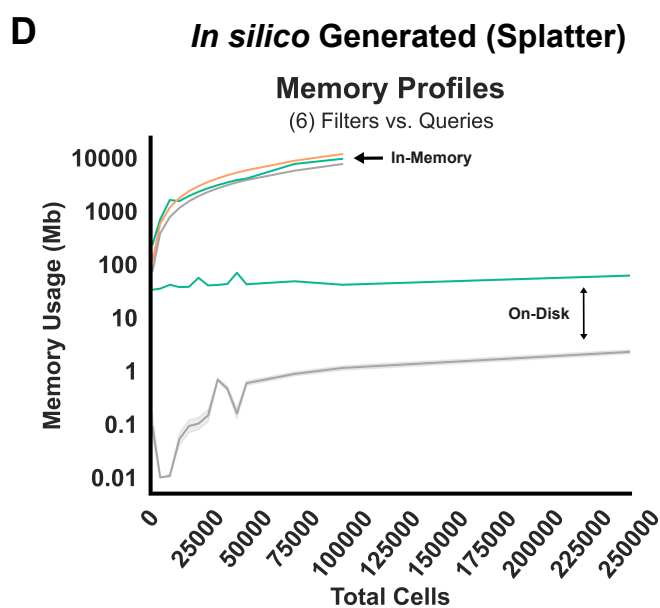

Supplement: vbaf105_Supplementary_Data [file vbaf105_supplementary_data.zip › Supplemental 2.pdf]
